# Development and evaluation of a patient passport to promote self-management in patients with heart diseases

**DOI:** 10.1186/s12913-019-4565-4

**Published:** 2019-10-21

**Authors:** Sabine Stamm-Balderjahn, Rebecca Faliniski, Susanne Rossek, Karla Spyra

**Affiliations:** Charité – Universitätsmedizin Berlin, corporate member of Freie Universität Berlin, Humboldt-Universität zu Berlin, and Berlin Institute of Health, Institute of Medical Sociology and Rehabilitation Science, Berlin, Germany, Charitéplatz 1, 10117 Berlin, Germany

**Keywords:** Cardiac rehabilitation, Patient passport, Patient self-management, Patient participation, eHealth

## Abstract

**Background:**

Patients with cardiovascular diseases (CVD) are treated over a long period of time by physicians and therapists from various institutions collaborating within a multidisciplinary team. Usually, medical records detailing the diagnoses and treatment regimens are long and extensive. Brief overviews of relevant diagnostic and treatment data in the form of a patient passport are currently missing in routine care for patients with CVD. This study aimed to develop and evaluate a patient passport (the Kardio-Pass) based on the needs of patients who had undergone cardiac rehabilitation, and of healthcare professionals.

**Methods:**

A mixed method design was adopted consisting of an explorative qualitative phase followed by a quantitative evaluation phase. Interviews with patients and experts were conducted to develop the Kardio-Pass. CVD rehabilitees (*N* = 150) were asked to evaluate the passport using a semi-standardized written questionnaire.

**Results:**

Patients and experts who were interviewed in the qualitative study phase considered the following passport contents to be particularly important: documentation of findings and diagnoses, cardiac diagnostics and intervention, medication plan, risk factors for heart disease, signs of a heart attack and what to do in an emergency. During the evaluation phase, 93 rehabilitees (response rate: 62%) completed the questionnaire. The Kardio-Pass achieved high overall approval: All respondents considered the information contained in the passport to be trustworthy. The professionalism and the design of the passport were rated very highly by 93 and 92% of participants, respectively. Use of the Kardio-Pass prompted 53% of participants to regularly attend follow-up appointments. The most common reasons for non-use were a lack of support from the attending doctor, failure by the patient to make entries in the passport, and loss of the passport.

**Conclusions:**

By documenting the course of cardiac diseases, the patient passport pools all medical data–from diagnosis to treatment and aftercare–in a concise manner. Rehabilitees who used the cardiac passport rated it as a helpful tool for documenting follow-up data. However, with regard to this explorative study there is a need for further research, particularly on whether the patient passport can improve heart patient care.

## Background

Coronary heart disease (CHD) was the most common type of heart disease in 2016, killing more than nine million people worldwide. It has claimed more lives than any other disease over the last 15 years [1]. Any strategy to effectively reduce risk factors involved in the progression of cardiovascular diseases must include the individual patients and their respective care structures. Active patient participation and continuous input from the patient should characterize the care process [2]. Therefore, in addition to prevention, treatment and rehabilitation, a patient-centred care process is also key.

In Germany, a differentiated healthcare framework is in place to help patients achieve occupational, domestic and social reintegration after acute treatment of cardiovascular events such as myocardial infarction (MI). One of the main goals of cardiac rehabilitation (CR, usually a three-week intervention) involves giving patients the skills to competently and independently cope with their disease so that, despite potential limitations, they can continue to work and remain a functional member of their family and society to the fullest extent possible. Chronic coronary heart disease (ICD I25) is the most common reason for CR in Germany, accounting for 37.9% of CR cases. Acute myocardial infarction (ICD I21) is the second most common diagnosis, accounting for 21.8% of cases. The remaining cases are due to other heart diseases such as cardiomyopathies and valvular heart diseases [3]. Therefore, more than half of all CR patients have chronic coronary artery disease. Efficient secondary care is crucial to helping them maintain and build on the successful reduction of cardiac risk factors achieved during rehabilitation.

Studies have shown that the progress made during phase III rehabilitation is largely lost if rehabilitees do not receive specific secondary care measures for long-term support [4]. Secondary care programmes that aim to initiate lasting lifestyle changes are only partially effective [5]. The short duration of these interventions is the most likely reason for their limited success. The long-term intervention strategy used in the GOSPEL study [6] achieved a marked improvement in lifestyle habits (e.g., exercise, diet and psychosocial stress) and in drug prescriptions for secondary prevention up to 3 years after rehabilitation following myocardial infarction. A significant reduction in cardiovascular mortality, nonfatal MI, nonfatal stroke, and cardiac death plus nonfatal myocardial infarction was also observed. In the intervention group, cardiac rehabilitation sessions were first conducted once a month and then semi-annually. Each session consisted of aerobic exercise (30 min), comprehensive lifestyle and risk factor counselling (at least 60 min) and reinforcement of preventive measures (30 min). The SEKONA study [7] is another successful long-term secondary prevention programme. It was based on telephone consultations that began at monthly intervals and continued on a quarterly basis over a period of 3 years. Patients in the intervention group showed better three-year risk profile outcomes than those in the control group.

The human and financial resources needed for these kinds of intervention programmes are so vast that they are generally not feasible in routine healthcare practice. Consequently, researchers should look for and investigate alternatives that place greater focus on patient self-management. A patient passport that transfers information to secondary healthcare professionals and increases the motivation of rehabilitees while supporting and stabilizing the lifestyle-change strategies learned during rehabilitation is suitable and could be used for this purpose [8]. Special patient passports already exist for some chronic diseases such as diabetes [9] and chronic inflammatory bowel disease [10]. A passport is available for cancer patients with brain tumours [11] and ENT tumours [12]. Passports for patients with cardiovascular disorders such as heart failure [13] and coronary heart disease [14] have also been described. However, none of these passports contain indications for active patient participation, such as entering laboratory and vital parameters oneself.

This study aimed to develop and evaluate a patient passport for standardized long-term care that would enable comprehensive and continuous interdisciplinary documentation of care for heart disease patients. The passport was to be based on the needs of heart patients and healthcare professionals, as well as on medical treatment recommendations and therapeutic guidelines for cardiovascular diseases [15]. Space was to be dedicated to secondary prevention measures for achieving positive behavioural changes in physical activity, diet, and abstinence from smoking and tobacco use. Theory-led strategies for successful behavioural change were to be based on the Health Action Process Approach (goal-setting, action planning, overcoming barriers and documenting success) [16].

## Methods

The mixed method design consisted of an exploratory qualitative phase followed by a quantitative evaluation phase.

### Qualitative study phase

The qualitative study phase was mainly exploratory in nature and provided initial insight into the new thematic field [17]. The exploratory part of the study was designed to generate the results needed to better structure the issue and establish a sound basis for developing the patient passport. Guided interviews and focus groups were conducted in order to identify ideas, wishes and needs with regard to the content, design and format of the passport. The aim of the focus groups was to acquire important information from potential users about the passport’s usability and acceptability. Because visual and haptic aspects will be important for the future use of the passport, we wanted to gather as many opinions as possible on this subject. In contrast to the focus groups, the interviews with experts (physicians, therapists and experienced rehabilitees) aimed to gain insights into what content the passport should include. All of the participants were asked to comment on a future digital version of the passport.

### Participants/recruitment

Eleven patient interviews, four focus groups with between three and seven participants, and ten expert interviews with physicians and therapists (cardiologists, general practitioners and sports therapists) were conducted. The patients were recruited from two German rehabilitation centres. The focus groups were composed of outpatient heart groups. The experts were recruited from two rehabilitation centres and from outpatient medical practices. Recruitment was discontinued once there was sufficient evidence that further data collection would lead to similar results (data saturation).

### Data analysis

The guideline-based interviews were conducted by topic and recorded using a digital audio recorder. We systematically analysed the recorded and transcribed interview data using Mayring’s qualitative content analysis [18] and the software MAXQDA 10 for manual inductive coding [19]. Two researchers inductively developed a coding system by independently reading the text material and combining their results according to themes and categories that emerged from the data. After discussing and mutually agreeing a coding frame, they coded all the texts individually. The researchers then reread the transcripts to identify any additional codes and potentially revise the frame.

### Quantitative study phase

#### Participants/recruitment

The study included cardiac rehabilitation patients (*N* = 150) who had a confirmed diagnosis of CHD, either with or without a percutaneous coronary intervention or bypass surgery or heart failure. The patients ranged from 18 to 80 years of age and were recruited from five German rehabilitation centres.

#### Questionnaire

The patients were asked to evaluate the Kardio-Pass using a semi-standardized written questionnaire that was sent to them 8 months after they had received the passport. The questionnaire was developed for the purpose of this study. The original German version was translated into English and is provided as Additional file 1. The questionnaire contained elements and information from existing instruments that measure indicators of usefulness and satisfaction [14, 20, 21]. In addition to closed questions, the questionnaire included open questions that allowed the respondents to provide more detailed answers to particular items. The closed questions asked respondents to rate their approval on a four-point scale: “strongly agree”, “agree”, “disagree” or “strongly disagree”. Where the rehabilitees specified a frequency of events, they used a five-point scale: “never”, “rarely”, “sometimes”, “often” or “always”.

The content of the questionnaire was structured in the same way as the patient passport, in which one section normally extends over two pages. Table 1 shows the individual sections of the passport and the patients’ evaluation line by line. For each section, patients were asked whether the language was understandable (comprehensibility) and, if applicable, whether the information and recommendations provided were useful. Overall approval of the passport was rated on a German school-grade scale of 1 (best) to 6 (worst). Finally, the rehabilitees were asked if they could imagine using the passport as a smartphone application. Rehabilitees who did not use the Kardio-Pass were asked to indicate the reasons for non-use. Data on the sociodemographic characteristics of the rehabilitees (gender, marital status, education and employment status) were also collected.

**Table 1:** Patient assessment of the topical sections in the Kardio-Pass

Topical section	Item(s) assessed	Strongly disagree % (n)	Disagree % (n)	Agree % (n)	Strongly agree % (n)
Instructions for use, table of contents, patient data, allergies, emergency contact, coagulation management	Comprehensibility			33.3 (21)	66.7 (42)
Doctors and institutions providing treatment (six stamp fields), overview of follow-up appointments	Comprehensibility	1.7 (1)	5.1 (3)	28.8 (17)	64.4 (38)
Cardiac diagnosis, concomitant diagnoses, stent intervention(s) and location(s), echocardiography findings, image of coronary arteries	Comprehensibility		14.3 (9)	34.9 (22)	50.8 (32)
	Usefulness of image of coronary arteries		14.5 (9)	35.5 (22)	50.0 (31)
Cardiovascular risk profile over time (body weight, waist circumference, smoking status, blood pressure, blood glucose, HbA1c, serum lipids)	Comprehensibility		4.7 (3)	40.6 (26)	54.7 (35)
Blood pressure and heart rate monitoring values, option for graphical display	Comprehensibility		3.3 (2)	26.2 (16)	70.5 (43)
Medications and dosage schedule (medication plan)	Comprehensibility			33.3 (21)	66.7 (42)
Information regarding the time after the rehabilitation intervention (e.g., advice on participation in a heart group, nutrition and relaxation courses)	Comprehensibility		1.6 (1)	36.1 (22)	62.3 (38)
	Usefulness of information		3.3 (2)	36.1 (22)	60.7 (37)
Heart group participation (place, time, training frequencies), intensified aftercare courses (place, time)	Comprehensibility		3.9 (2)	33.3 (17)	62.7 (32)
Physical activities (recommendations, objectives, action planning), diary for physical activities	Comprehensibility		3.4 (2)	39.0 (23)	57.6 (34)
	Usefulness of recommendations		11.5 (7)	32.8 (20)	55.7 (34)
Schematic illustration of heart attack signs, information about what to do in an emergency	Usefulness of information		3.1 (2)	23.1 (15)	73.8 (48)

#### Data analysis

Statistical analysis of the standardized questions consisted mainly of descriptive statistics (e.g., frequency distribution, chi-square test). Binary logistic regression was used to test for associations between certain variables. The statistical analysis was performed using SPSS Statistics Version 22. Responses to the open questions were evaluated by content analysis.

#### Ethical considerations

Before starting the present study, a study approval request (EA1/066/15) was submitted to the ethics committee of Charité – Universitätsmedizin Berlin. It was granted on 31 March 2015. Participation in the study was voluntary. Informed written consent was obtained from all patients when enrolling them in the study.

## Results

### Qualitative analysis

#### Participants

The sample of patients interviewed included five women (45%) and six men (55%). They had a mean age of 61 ± 11.1 years. Of the 20 focus group participants, four (20%) were female and 16 (80%) were male. The mean age was 67.6 ± 8.5 years. Of the ten experts interviewed, six were male (60%) and four were female (40%). On average, they had practiced medicine for 20.8 ± 14.1 years.

#### Themes

Five main themes reflecting the patients’ views on the development of a patient passport were identified from the interview data: (i) knowledge and usefulness of a patient passport for cardiological patients; (ii) management of the passport; (iii) format and visual representation; (iv) medical and behavioural content; (v) future digitalization of the passport.

Regarding (i), most of the interviewees had never heard of a cardiac passport. Just two patients, both working women, knew about them and had used one before. Nearly all patients surveyed were in favour of developing a patient passport. One of the patients summed up what a cardiac passport would mean to him as follows:

“Well, personally, I would *very* much like to have a patient passport like that. Because with that format, all the information needed in an emergency would be in the passport, and we don’t have anything like that now.”

Half of the experts were familiar with patient passports, but very few knew of a comprehensive passport for interdisciplinary documentation of care. In reference to this problem, one expert stated:

“Every now and then a patient will pull out one of those things and you’re supposed to write something in it. Most patients… well, we have Marcumar ID cards, and now there are a couple of stent passports. I saw that recently. But to my knowledge, no true cardiac passport exists, generally speaking.”

By and large, the experts agreed that a comprehensive passport for cardiac patients would be important and useful. Only one expert noted that the quality of the content and the level of patient acceptance would ultimately determine whether patients would actually use a passport.

Regarding (ii), when asked who should be allowed to make entries in the cardiac passport, most of the focus group participants thought that this should be done by medical personnel only. One participant voiced the following opinion:

“You just have to decide *who* is leading the passport. Do you enter it yourself? […] Then *everyone* would have to go along with it. Otherwise, if it’s not updated, it can cause harm.”

By contrast, most of the patients and experts were in favour of patients making their own entries.

Regarding (iii), the main topic of discussion in the focus groups concerned passport design preferences (size, image and entry format). When asked about size options, the focus group participants preferred a DIN A6 format (4.13 × 5.83 in.). In terms of the illustration of the heart, most participants preferred the coronary artery image that can be used to document stent location. Only a few participants preferred the schematic diagram of the heart anatomy. As for the experts, they favoured a DIN A6 format. They also felt that the patient’s personal data should not appear on the front cover, but rather on the first page of the passport if possible. Like some of the patients, the experts thought that emergency contact information should be included in the passport.

Regarding (iv), the interviewed patients felt that the following content was essential: documentation of medications, diagnoses and medical findings, emergency contact information, and outpatient examination and test results. Most of the experts’ responses about the Kardio-Pass content concerned the documentation of diagnostic measures, cardiac interventions, medications, diagnoses and risk factors. They also felt that it would be beneficial if the passport provided reminders for preventive medical check-ups and documented laboratory values and medications over time.

Regarding (v), most of the experts were open to the idea of making the passport available as a digital healthcare app. However, some raised objections. One cardiologist commented as follows:

“It depends on the age of the users. The younger patients can certainly handle an app well. But patients should be able to choose which form they want to use.”

Most of the patients rejected the idea of a mobile health app, but younger and employed patients expressed fewer reservations. Moreover, some patients raised concerns about data protection and privacy. In the focus groups, opinions were divided on whether the passport should be available as an application. Approximately half of the participants voted in favour of an app, nearly a quarter were against it, and the remaining quarter abstained. Among the supporters and opponents of the passport app, some envisaged the app being used more by the younger generation and in the future. This opinion was held by a fifth of the focus group participants. Most of the participants who rejected the app indicated that data protection concerns were their main reason for doing so. One person fundamentally rejected the use of any type of modern technology.

The knowledge gained from the expert and patient interviews and from the focus group discussions provided the basis for developing the patient passport. The Kardio-Pass currently contains 24 pages, including the front and back cover. It is available in a DIN C6 format (4.49 × 6.38 in.). A PDF version of the Kardio-Pass is provided in Additional file 2.

### Quantitative analysis

#### Participants

The quantitative analysis included 150 rehabilitees with a median age of 59.2 ± 9.1 years (range: 40 to 78 years). Of those surveyed, 82.8% were male and 17.2% were female. A total of 64.8% were employed, with 91.5% working full-time. Most rehabilitees (72.2%) were married or lived with a partner. The rest were either single (13.3%), divorced (11.1%) or widowed (3.3%). The majority (52.2%) had an intermediate high school diploma (10 years), 26.7% had a higher level of high school education, and 21.1% had a lower level.

Ninety-three rehabilitees completed and returned the questionnaire, yielding a response rate of 62%. Sixty-seven (72%) of the respondents reported using the Kardio-Pass. Twenty-six of those who did not use the passport indicated their reasons for non-use.

#### Findings

The Kardio-Pass is divided into individual topical sections (e.g., diagnosis, cardiovascular risk profile, medication plan). The questionnaire followed this structure. Table 1 shows the user assessments of the comprehensibility and usefulness of the passport content.

Fifty-three percent (*n* = 35) of respondents stated that using the Kardio-Pass had prompted them to attend follow-up appointments regularly, while 57.6% (*n* = 34) found that the passport was a helpful support tool for communicating with doctors. Twenty-one percent (*n* = 13) felt that the Kardio-Pass had improved their doctor-patient relationship, and 88.2% (*n* = 56) said that it gave them an increased sense of security in various situations (when out and about, in an emergency or when taking medications). Nearly 60% (*n* = 39) took advantage of the possibility to enter information in the passport themselves (e.g., follow-up appointment dates and monitoring values for risk parameters), and 37.8% (*n* = 23) used the passport to log physical activities.

The rehabilitees were also asked to rate their general impression of the Kardio-Pass in terms of the trustworthiness of the information and its professional impression, design and format/size (Table 2). The average rating was 2.1 on a scale of 1 (best) to 6 (worst).

**Table 2:** Patients’ general impression of the Kardio-Pass

General impression of the Kardio-Pass	Strongly disagree % (n)	Disagree % (n)	Agree % (n)	Strongly agree % (n)
Trustworthy information			29.2 (19)	70.8 (46)
Professional impression		6.4 (4)	22.6 (14)	71.0 (44)
Good design (colour, layout)		8.1 (5)	45.2 (28)	46.8 (29)
Good format/size	11.1 (7)	15.9 (10)	34.9 (22)	38.1 (24)

In total, 47.8% (*n* = 32) of the rehabilitees said they could imagine using the Kardio-Pass as a mobile app in the future. This result was not affected by age or gender. Among those who could not imagine using it as an app, the most commonly mentioned reasons were not owning a smartphone, a lack of experience with apps, and data privacy concerns.

Twenty-eight percent (*n* = 26) of rehabilitees reported that they had not used the Kardio-Pass after receiving it. Passport users and non-users did not differ significantly in terms of gender, marital status and occupational status. Multivariate analysis showed higher rates of passport use among older rehabilitees (odds ratio: 1.07; *p* = 0.04) and those with higher education levels (odds ratio: 2.52; *p* = 0.02). The three most common reasons for non-use were a lack of support from the attending doctor, failure of the patient to make entries in the passport, and loss of the passport.

## Discussion

### Key findings

The Kardio-Pass was developed with cardiac rehabilitees and with medical experts involved in the co-treatment and secondary care of heart disease patients. The passport enables comprehensive, interdisciplinary and continuous documentation of the course of disease. In the second phase of the study, CHD rehabilitees tested the suitability and practicality of the Kardio-Pass for 8 months. They had the option of entering data in their passports themselves to document the course of their disease. The surveyed users had a positive general impression of the passport (Table 2). All users rated the information contained in the passport as trustworthy, 93.6% felt the passport made a professional impression and 92% liked the design. However, 27% of users criticized the format and design, and most thought that it was too large.

### Interpretation

Detailed analysis of approval ratings for the individual topical sections revealed that, in terms of comprehensibility, only 85.7% of respondents approved of the “Diagnoses and Findings” section (Table 1, Row 4). All other sections achieved approval ratings above 90%. “Diagnoses and Findings” mainly contains technical terms used to describe cardiac diagnoses, concomitant diagnoses and echocardiography findings. The lower approval ratings suggest that the patients lacked the knowledge needed to understand the content.

Twenty-eight percent (*N* = 26) of the patients surveyed reported that they did not use the Kardio-Pass after receiving it. Lack of support from the attending doctor was the most common reason for non-use. From the perspective of the rehabilitees, this finding might be symptomatic for follow-up rehabilitation. In the final report on the study *Aftercare: wishes and reality from the perspective of rehabilitees with musculoskeletal diseases*, the authors conclude that rehabilitees need professional support during aftercare in order to help them become competent in self-management [22]. In addition, patients should receive professional support as they continue to use the Kardio-Pass because research has shown that including patients in the documentation process strengthens patient participation and personal responsibility [23]. A failure to recognize this fact, along with time constraints, might be why cardiac rehabilitees do not receive the necessary support from the doctors providing follow-up care. However, the passport has the potential to improve doctor-patient relationships. A fifth of the rehabilitees surveyed in our study confirmed this. In turn, a good doctor-patient relationship is the basis for effective patient self-management and can lead to better health outcomes [24, 25].

Our analysis of the “Physical Activities, Planning and Diary” section of the Kardio-Pass showed a gap between the patients’ perceptions of the usefulness of the recommendations and their actual use of the documentation materials. The Kardio-Pass provides guideline-based physical activity recommendations [26] and gives concrete tips for successfully implementing them (setting goals, planning action and monitoring success) based on the Health Action Process Approach (HAPA) [27]. Although 88.5% of the rehabilitees rated the physical activity recommendations as useful, most of them (62.3%) never actually used the diary to document their daily or weekly physical activities. Obviously, these rehabilitees failed to bridge the intention-behaviour gap in which intended and actual behaviours do not coincide, as described by Sheeran [28]. Space limitations in the Kardio-Pass (there is only room for 14 days of entries) could also be a reason for non-use of the diary.

Scope for comparing the results on passport acceptance and user analysis from the present study with those from other studies is limited. To our knowledge, only one other study exists in which researchers investigated the acceptance and use of a patient passport for secondary prevention of heart disease using a similar methodology. In that study, by Völler et al. [14], the passport was mainly used to document changes in physiological protective factors and risk factors over time. By contrast, our study mainly focused on user assessments of the passport by cardiac rehabilitees.

Völler et al. [14] issued 437 patients who had a confirmed CHD diagnosis with a health passport before they were discharged from inpatient rehabilitation. The rehabilitees were asked to carry the passport for 1 year and then return it to the study centre. The analysis revealed that only 44% of the patients had used the passport regularly over the course of the year. Consequently, the authors concluded that acceptance of the passport was low. In our study, 72% of cardiac rehabilitees used the Kardio-Pass regularly. However, the two studies produced similar findings regarding the demographic characteristics of passport users and non-users. Like Völler et al. [14], we found that older participants and those with higher levels of education tended to use the passport more often. Gender-specific differences were not detected in either of the two studies.

British researchers investigated the acceptance of a patient passport for asthma management in a clinical study of patients with severe asthma [29]. The aim of introducing the passport was to save lives by ensuring that patients could access emergency services when needed, and to help healthcare professionals deliver timely, appropriate and individualized emergency treatment. The investigators issued the passport to 15 asthma patients, seven of whom used it 15 times. This was perceived as helpful because it made the patients feel less insecure and apprehensive. Likewise, 88.2% of the patients surveyed in our study stated that the passport gave them a greater sense of security in various situations (when out and about, in an emergency or when taking medications).

### Future prospects

The most obvious disadvantage of our Kardio-Pass is the limited space available for logging protective and risk factor measurements (such as blood pressure, heart rate, weight, blood glucose and cholesterol levels), updating information on medications and activity plans, noting scheduled doctor appointments and documenting test findings (e.g., echocardiography, training frequency). Therefore, the study included a question about the possibility of making the Kardio-Pass into a mobile application. Nearly half (47.8%) of the respondents indicated that they could imagine using such an app. A representative survey carried out by Bitkom (the German Association for IT, Telecommunications and New Media) in May 2017 produced similar results. It showed that almost half (45%) of Germany’s smartphone users could imagine using health apps in the future, and that nearly one in two smartphone owners already used a mobile health app [30].

In principle, mobile health apps offer scope for patient participation and can support different phases of care processes within the healthcare system [31]. Preliminary studies show that health apps can be of particular benefit to patients with cardiovascular diseases. Development of these applications should be based on theoretical models for explaining behavioural changes. It is useful to include video game elements, reward systems and social media elements in the apps [32]. In light of this, a mobile Kardio-Pass application (the Kardio-Pass app) will be developed in the future on the basis of the user survey results presented in this study.

### Limitations

This study has some limitations. For example, the research is based exclusively on observations and does not provide the evidence needed to draw conclusions regarding effectiveness. A controlled trial is needed to determine whether and which clinical parameters are positively influenced by maintaining a cardiac passport. Furthermore, the response rate was quite low (62%), which could lead to bias in the results. A comparison of respondents with non-respondents showed no differences in age (*p* = 0.46) and gender (*p* = 0.53). No bias was detectable for these two characteristics. However, other factors not considered in this study (e.g., education, profession) might also influence the response rate. Furthermore, a distortion of the qualitative results is possible because the focus groups consisted mainly of men.

## Conclusions

Doctors, therapists and patients were included in the Kardio-Pass development process. To our knowledge, it is the first cardiac passport of its kind. The Kardio-Pass is a standardized instrument for providing long-term care to heart disease patients. It makes it possible to consolidate all of a patient’s medical data—from diagnosis and primary treatment to secondary care—in a clear and concise manner. The passport allows everyone involved in the treatment process to obtain an up-to-date and comprehensive picture of the current status at any time. Rehabilitees who used the Kardio-Pass rated it as a helpful tool for documenting their follow-up. Plans are in place to develop a Kardio-Pass mobile app. Further useful functions (documentation of follow-up data without time limits, and reminders for medications and appointments) and health-related information (information about cardiac risk factors and a glossary) could be integrated into the app. Ultimately, patients will be able to decide whether they want to use the mobile app or the hard copy of the patient passport. To make the Kardio-Pass easily accessible for rehabilitees, it should be available to all interested rehabilitation facilities with cardiological departments.

## Supplementary information

**Additional file 1.** English translation of the questionnaire used in this study.

**Additional file 2.** Original, printable version of the Kardio-Pass.

## Data Availability

The dataset used and analysed during the study is available from the corresponding author on request.

## Abbreviations

CHD: Coronary heart disease
CR: Cardiac rehabilitation
CVD: Cardiovascular disease
ENT: Ear, nose and throat
HbA1c: Glycated haemoglobin
ICD: International Classification of Diseases
MI: Myocardial infarction
