# Correction to: Development and evaluation of a patient passport to promote self-management in patients with heart diseases

**DOI:** 10.1186/s12913-020-05545-3

**Published:** 2020-07-22

**Authors:** Sabine Stamm-Balderjahn, Rebecca Falinski, Susanne Rossek, Karla Spyra

**Affiliations:** Berlin Institute of Health, Institute of Medical Sociology and Rehabilitation Science, Charité – Universitätsmedizin Berlin, Freie Universität Berlin, Humboldt-Universität zu Berlin, Charitéplatz 1, 10117 Berlin, Germany

**Correction to: BMC Health Serv Res 19, 716 (2019)**

**https://doi.org/10.1186/s12913-019-4565-4**

Following publication of the original article [1], the authors identified an error in the author name of Rebecca Falinski.

The incorrect author name is: Rebecca Faliniski.

The correct author name is: Rebecca Falinski.

The author group has been updated above.
