# Supplementary material for: Development and evaluation of a patient passport to promote self-management in patients with heart diseases
Source: BMC Health Serv Res. 2019 Oct 21;19:716. doi: 10.1186/s12913-019-4565-4 (PMC6805613; doi:10.1186/s12913-019-4565-4)
Supplement: Supplementary file 1 — Additional file 1. English translation of the questionnaire used in this study. [file 12913_2019_4565_MOESM1_ESM.pdf]

CHARITÉ
UNIVERSITÄTSMEDIZIN BERLIN

Kardio-Pass questionnaire

- ✓ With this questionnaire we would like to know your opinion on the usefulness of the Kardio-Pass.
- ✓ To answer the questions, please take your Kardio-Pass.
- ✓ If you did not use the Kardio-Pass, please start on page 7 with question 40.

Study-ID:			

CHARITÉ
UNIVERSITÄTSMEDIZIN BERLIN

Questions on the individual sections of the Kardio-Pass

**Instruction for use | Patient data (pages 2-3)**					
Please tick <u>one answer</u> for each statement.		Strongly disagree	Disagree	Agree	Strongly agree
1.	I find the design clear.	O	O	O	O
2.	The content is easy to understand.	O	O	O	O
3.	Did you enter the patient data yourself (personal data, allergies, coagulation management, emergency contact)?				
	Yes				O
	No				O

**Doctors and institutions providing treatment | Overview of follow-up appointments (pages 4-5)**					
Please tick <u>one answer</u> for each statement.		Strongly disagree	Disagree	Agree	Strongly agree
4.	I find the design clear.	O	O	O	O
5.	The content is easy to understand.	O	O	O	O
6.	How often did you enter appointments in the appointment overview? (page 5)? Please tick <u>one answer</u>.				

	Never	Rarely	Occasionally	Often	Always
	O	O	O	O	O

**Cardiac diagnosis (pages 6-7)**					
Please tick <u>one answer</u> for each statement.		Strongly disagree	Disagree	Agree	Strongly agree
7.	I find the design clear.	O	O	O	O
8.	The content is easy to understand.	O	O	O	O
9.	I find the imaging of the coronary arteries helpful.	O	O	O	O
10.	Did you make the entries for this section yourself (cardiac diagnosis, intervention, coronary arteries, concomitant diagnoses, cardiac echocardiography findings)?				
	Yes				O
	No				O

Cardiovascular risk profile (pages 8-9)					
Please tick one answer for each statement.		Strongly disagree	Disagree	Agree	Strongly agree
11.	I find the design clear.	O	O	O	O
12.	The content is easy to understand.	O	O	O	O

13.	How often did you enter your values in the cardiovascular risk profile (pages 8-9)? Please tick one answer.				
	Never	Rarely	Occasionally	Often	Always
	O	O	O	O	O

Blood pressure and heart rate monitoring values (pages 10-11)					
Please tick one answer for each statement.		Strongly disagree	Disagree	Agree	Strongly agree
14.	I find the design clear.	O	O	O	O
15.	The content is easy to understand.	O	O	O	O

16.	How often did you enter the values for blood pressure and pulse (pages 10-11)? Please tick one answer.				
	Daily	Serveral times a week	One to three times a month	One to three times a half year	Never
	O	O	O	O	O

Medications and dosage schedule (pages 12-13)					
Please tick one answer for each statement.		Strongly disagree	Disagree	Agree	Strongly agree
17.	I find the design clear.	O	O	O	O
18.	The content is easy to understand.	O	O	O	O

19.	Did you make any entries on your medications (medication, dose, schedule)? Please tick one answer.	
	Yes	O
	No	O

Information for the time after rehabilitation (pages 14-15)					
Please tick one answer for each statement.		Strongly disagree	Disagree	Agree	Strongly agree
20.	I find the design clear.	O	O	O	O
21.	The content is easy to understand.	O	O	O	O
22.	The individual notes are helpful.	O	O	O	O

Participation in a heart group (pages 16-17)					
Please tick one answer for each statement.		Strongly disagree	Disagree	Agree	Strongly agree
23.	I find the design clear.	O	O	O	O
24.	The content is easy to understand.	O	O	O	O
25.	Did you make the entries for your heart group participation yourself (page 16)? Please tick one answer.				
	Yes				O
	No				O
	I do not participate in any heart group.				O

Physical activities | Diary for physical activities (pages 18-21)					
Please tick one answer for each statement.		Strongly disagree	Disagree	Agree	Strongly agree
26.	I find the design clear.	O	O	O	O
27.	The content is easy to understand.	O	O	O	O
28.	The recommendations for regular physical activities are helpful.	O	O	O	O
29.	How often did you use your diary for physical activities (pages 20-21)? Please tick one answer.				
	Daily	Serveral times a week	One to three times a month	One to three times a half year	Never
	O	O	O	O	O

Heart attack signs (Back of the Kardio-Pass)					
Please tick one answer.		Strongly disagree	Disagree	Agree	Strongly agree
30.	I find the list of signs of a heart attack and the graphic illustration helpful.	O	O	O	O

Further questions on the Kardio-Pass

31.	Did the use of the passport motivate you to regularly attend your follow-up appointments? Please tick one answer.	
	Yes	O
	Rather yes	O
	Rather no	O
	No	O

32.	In which situations does the passport give you a feeling of security and in which situations was it perceived as helpful? Several answers are possible.		
	a) In the following situations I feel safe with the passport:	When I'm out and about.	O
		In case of emergency (emergency contacts, medication information).	O
		When I take my medication.	O
		Other situations (please specify):	
		The passport didn't give me any sense of security.	O
	b) The passport is useful in the following situations:	Scheduling coordination.	O
		Communication with the doctor (e.g. asking questions).	O
		Planning my physical activities.	O
		In nutrition.	O
		Other situations (please specify):	
		I didn't find the passport helpful.	O

33.	Do you feel that the use of the passport has affected the relationship with your attending doctor? Please tick one answer.	
	Improved	O
	Remained the same	O
	Deteriorated	O

General questions about the content, structure and design of the Kardio-Pass

**The following questions relate to the general impression of the passport.**					
Please tick one answer for each statement.		Strongly disagree	Disagree	Agree	Strongly agree
34.	I think the information is trustworthy.	O	O	O	O
35.	The passport makes a professional impression.	O	O	O	O
36.	I like the design (color, layout) of the passport.	O	O	O	O
37.	I like the format and size of the passport.	O	O	O	O

38.	Please rate the passport with a school grade (1 for "very good" and 6 for "insufficient"). Please tick one answer.					
	1	2	3	4	5	6
	O	O	O	O	O	O

39.	Instead of the print-version of the passport, can you imagine using a smartphone app with information and options for personal notes on your coronary heart disease in the future? Please tick one answer.	
	Yes	O
	Rather yes	O
	Rather no	O
	No	O
	If "rather no" or "no": why not? Please state your reasons.	

CHARITÉ
UNIVERSITÄTSMEDIZIN BERLIN

→ Please continue with question **41.**

Reasons for "non-use" of the Kardio-Pass

40.	If you did not enter anything in the Kardio-Pass, please state your reasons. Several answers are possible.	
	I didn't have any time.	O
	I didn't understand what should be entered.	O
	I had no desire.	O
	There was no support from my relatives.	O
	There was a lack of support from my attending doctor.	O
	I lost the passport.	O
	I forgot to make entries.	O

Your personal data

41.	Please specify your gender.	
	Male	O
	Female	O

42.	Please state your marital status. Please tick one answer.	
	Single	O
	Married or in partnership	O
	Devorced	O
	Widowed	O

43.	What is your highest school qualification? Please tick one answer.	
	Secondary school	O
	Intermediate school	O
	Higher school	O

44.	What is your professional situation? Please tick one answer.	
	Working full-time	O
	Working part-time	O
	Not working	O

Thank you very much for taking the time to answer the questions!
