# Supplementary material for: Development and evaluation of a patient passport to promote self-management in patients with heart diseases
Source: BMC Health Serv Res. 2019 Oct 21;19:716. doi: 10.1186/s12913-019-4565-4 (PMC6805613; doi:10.1186/s12913-019-4565-4)
Supplement: Supplementary file 2 — Additional file 2. Original, printable version of the Kardio-Pass. [file 12913_2019_4565_MOESM2_ESM.pdf]

Kardio-Pass

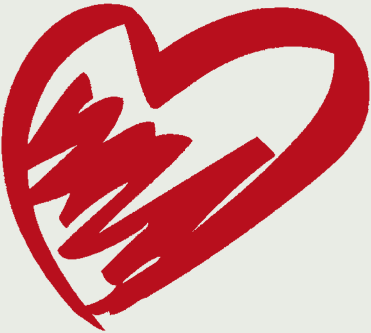

Studiennummer | | |

Liebe Patientin, lieber Patient,

Ihr persönlicher Kardio-Pass unterstützt Sie und Ihre behandelnden Ärzte bei der optimalen Behandlung Ihrer Herzerkrankung.

Als ständiger Begleiter enthält er unter anderem wichtige Angaben zu Ihren Diagnosen, zu relevanten Untersuchungsergebnissen, Ihrem Herz-Kreislauf-Risiko-Profil, eine Auflistung Ihrer verordneten und einzunehmenden Medikamente sowie eine Übersicht zu Ihren Terminen.

Ferner finden Sie hilfreiche Tipps und Anregungen, die Ihnen das Leben mit Ihrer Erkrankung erleichtern und Ihre Gesundheit fördern.

Inhaltsverzeichnis

	Seite
Benutzerhinweise | Patientendaten	2 - 3
Behandelnde Ärzte und Kliniken | Terminübersicht	4 - 5
Diagnosen | Befunde Herz–Echokardiographie	6 - 7
Herz–Kreislauf–Risikoprofil | Persönliche Verlaufswerte	8 - 9
Verlaufswerte Blutdruck	10 - 11
Medikamentenübersicht | Einnahmeschema	12 - 13
Reha – und dann? | Das tut mir gut	14 - 15
Herzgruppe | Nachsorge „IRENA“	16 - 17
Planen von körperlichen Aktivitäten	18 - 19
Tagebuch für körperliche Aktivitäten	20 - 21
Persönliche Notizen | Impressum	22 - 23

Name: ....

Anschrift: ....

Telefon: ....

E-Mail: ....

Geburtsdatum: ....

Allergien

	Ja	Nein	Unbekannt
Röntgenkontrastmittel:	☐	☐	☐
Medikamente:	☐	☐	☐

....

....

....

Andere:	☐	☐	☐

....

....

....

Gerinnungsmanagement

	Ja	Nein	Unbekannt
(Medikamente siehe Seite 12–13)	☐	☐	☐

Im Notfall bitte benachrichtigen

Name: ....

Telefon: ....

Akut

Reha

Hausarzt

Ambulante Herzgruppe

Kardiologe

Entlassung aus der Reha

Datum: ....

Kontrolluntersuchung	Kontrolluntersuchung

Kardiale Diagnose

	Datum
Stabile Angina Pectoris:	....
Instabile Angina Pectoris:	....
Myokardinfarkt: ☐ STEMI	....
☐ Non-STEMI	....
Herzinsuffizienz – NYHA Klasse:	....

Intervention (mit Datum):

....

....

....

....

(z. B.: Stent/Segment; Bypass-OP; ICD-Träger; Herzklappen-OP)

Ihre Herzkranzgefäße

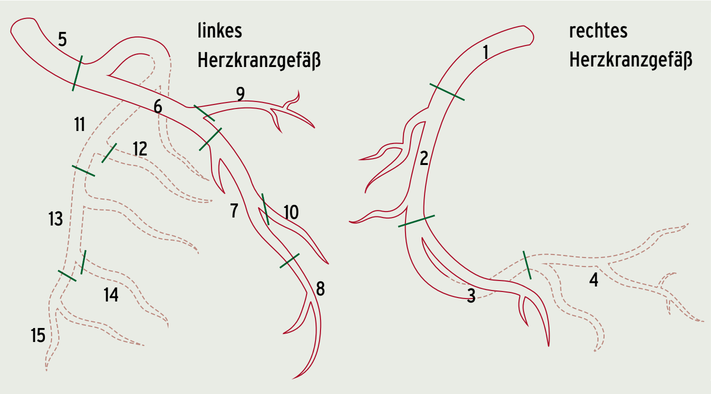

Begleitdiagnosen

....

....

....

....

....

....

....

....

....

Befunde Herz-Echokardiographie

Datum: ....

- ☐ EF: .... % IVS: .... mm
- ☐ LVEDD: .... mm LA: .... mm
- ☐ Klappen: ....
- ☐ Weiteres: ....

Datum: ....

- ☐ EF: .... % IVS: .... mm
- ☐ LVEDD: .... mm LA: .... mm
- ☐ Klappen: ....
- ☐ Weiteres: ....

Datum	Körperliche Aktivität* (Stunden/Woche)	Körpergewicht (kg)	Taillenumfang (cm)	Blutdruck* (mm Hg)
5.1.2016	4	89	72	138/81

* Weitere Möglickeiten zum Eintragen: Körperliche Aktivität Seite 20–21, Blutdruck Seite 10–11

Blutzucker (1 = mg/dl) (2 = mmol/l)	HbA1c (1 = %) (2 = mmol/mol)	HDL-Cholesterin (1 = mg/dl) (2 = mmol/l)	LDL-Cholesterin (1 = mg/dl) (2 = mmol/l)	Rauchstatus (Zigaretten/ Tag)
98 (1)	5,9 (1)	1,4 (2)	2,5 (2)	10

Beispielwerte

Datum	5.1.16	6.1.16	7.1.16				
Syst. BD	149	155	140				
Diast. BD	95	95	93				
Puls	86	84	80				

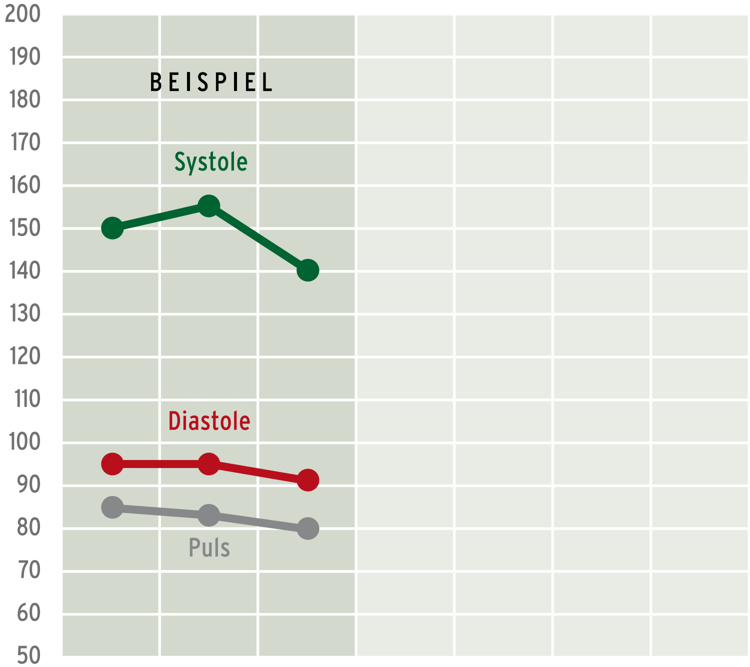

Notizen: ....

....

Notizen:

Diese Seiten können als Kopiervorlage genutzt werden oder im Downloadbereich des Onlineportals unter www.herzwegweiser.de heruntergeladen werden.

Medikamente	Dosis (mg)
**Blutdrucksenker** (z. B. Lopresor®, Delix®)	
**Cholesterinsenker** (z. B. Zocor®, Sortis®)	
**Gerinnungshemmer** (z. B. Marcumar®, Pradaxa®)	
**Plättchenhemmer** (z. B. Clopidogrel®, ASS®)	
Weitere Medikamente:	

morgens	mittags	abends	Einnahme bis (Datum)

Wie geht es weiter?

Um das Fortschreiten der Erkrankung zu verhindern, ist die Weiterführung einer gesunden Lebensweise von besonderer Bedeutung:

- **Körperliche Aktivität, gesunde Ernährung, Nichtrauchen und seelisches Wohlbefinden sind dabei wichtige Bausteine.**

Ziel der Rehabilitationsträger (z.B. der Deutschen Rentenversicherung oder der Krankenkassen) ist es, Sie dabei zu unterstützen. So haben Sie beispielsweise einen Anspruch auf Teilnahme am Rehabilitationssport innerhalb von **Herzgruppen** (siehe Seite 16). Die Kosten hierfür werden in der Regel für 90 Übungseinheiten übernommen, die Sie innerhalb von 24 Monaten absolvieren können. Es ist lediglich die Verordnung durch den behandelnden Arzt oder die Ärztin erforderlich.

Auch kann ein Austausch mit anderen Menschen, die sich in der gleichen Situation befinden, hilfreich sein. In **Selbsthilfegruppen** können Sie sich über Ihre Erkrankung informieren und über Ängste und Sorgen sprechen.

Auf dem Internetportal **www.herzwegweiser.de** finden Sie wohnortnahe Anbieter von Herzgruppen, Selbsthilfegruppen und weiteren Gesundheitskursen sowie ausführlichere Informationen zur Nachsorge.

Bei allen Fragen zur **beruflichen Wiedereingliederung** geben fachkundige Mitarbeiterinnen und Mitarbeiter der Deutschen Rentenversicherung zielgerichtete Informationen und stehen Ihnen mit Rat und Tat zur Seite:

- **www.deutsche-rentenversicherung.de**
  Servicetelefon: 0800 1000 4800

Körperliche Aktivität | Kein Medikament hat so viele positive Effekte auf den Körper wie regelmäßige Bewegung. Durch körperliche Aktivität können Sie sich vor koronarer Herzkrankheit schützen oder das Voranschreiten bremsen.

▶ **Deshalb: Bewegen Sie sich! Seien Sie aktiv!**

Auf den Seiten 18–19 finden Sie eine Anleitung zur Planung Ihrer körperlichen Aktivitäten.

Gesunde Ernährung | Eine gesunde Ernährungsweise (reichlich Gemüse, Obst und Fisch sowie wenig Fleisch und Wurst) wirkt sich günstig auf Ihr Herz und Ihr Gefäßsystem aus. Um eine nachhaltige Veränderung Ihres Essverhaltens zu erreichen, können Sie sich in Ernährungs- und Kochkursen durch Ernährungsberater begleiten lassen.

Nichtrauchen | Nicht zu rauchen ist das Beste, was Sie für Ihr Herz tun können. Oft ist es nicht einfach, mit dem Rauchen aufzuhören und wenn es Ihnen schwerfällt, können Sie sich unterstützen lassen. Es gibt vielfältige Möglichkeiten, dauerhaft ein rauchfreies Leben zu erreichen.

Entspannung/Stressmanagement | Die Reduzierung von Stressfaktoren ist eine wichtige Vorkehrung, um dem Fortschreiten der Erkrankung vorzubeugen. In Entspannungskursen können Sie Techniken erlernen, mit denen Sie innerlich zur Ruhe kommen oder sich Verspannungen lösen lassen, zum Beispiel autogenes Training, progressive Muskelentspannung (PMR), Yoga, Pilates, Tai-Chi oder Qigong.

▶ **Die Teilnahme an Tabakentwöhnungsprogrammen, Entspannungs- und Ernährungsberatungskursen wird von den Krankenkassen bezuschusst.**

Verein

Teilnahme seit wann? (Datum):

....

Meine Herzgruppentermine:

....

....

....

Trainingsfrequenz Datum: ....

Blutdruck in Ruhe: .... mmHg

HF in Ruhe: ....

Ergometrie: .... Watt (höchste belastete Leistungsstufe)

.... max. HF

.... mmHg (höchster RR)

Trainingsfrequenz Datum: ....

Blutdruck in Ruhe: .... mmHg

HF in Ruhe: ....

Ergometrie: .... Watt (höchste belastete Leistungsstufe)

.... max. HF

.... mmHg (höchster RR)

„IRENA“ ist ein multimodales Nachsorgeprogramm der Deutschen Rentenversicherung. Es kann im Anschluss an eine stationäre oder ganztägig ambulante Rehabilitationsmaßnahme vom Ärzteteam am Ende des Aufenthaltes in der Rehabilitationseinrichtung empfohlen werden. Der inhaltliche Rahmen wird durch das Ärzteteam empfohlen. Die Kosten werden für insgesamt 24 Termine übernommen und können innerhalb von sechs Monaten ab Entlassung aus der Rehabilitationseinrichtung wahrgenommen werden.

Zentrum

Beginn (Datum): ....

Uhrzeit: ....

☐ Mo ☐ Di ☐ Mi ☐ Do ☐ Fr ☐ Sa

▶ **Weitere Informationen auf www.herzwegweiser.de**

Sie finden dort umfassende Informationen für die Nachsorge, können bequem über die Suche wohnortnahe Anbieter von Gesundheitskursen (z. B. Herzgruppen, Entspannungskurse, etc.) in der Region Berlin-Brandenburg finden und gelangen zum Downloadbereich für die Zusatzmaterialen zum Kardio-Pass.

Empfehlungen für regelmäßige körperliche Aktivität

- **Ausdauerbelastung trainieren**
  mindestens dreimal pro Woche (besser täglich)
  mindestens 30 Minuten
  Sportarten, die sich besonders eignen: Flottes Gehen, Radfahren, Joggen, Nordic-Walking, Schwimmen, Skilanglauf
  sowie
- **Kraftausdauerübungen zum allgemeinen Muskelaufbau**
  zwei- bis dreimal pro Woche | 15 – 30 Minuten
  Hierfür eignen sich: Übungen mit dem elastischen Gymnastikband, Gerätetraining.

- Schritt 1: **Ziel setzen**

  Wie oft will ich mich pro Woche körperlich bewegen?

  Mein Ziel: .... h/Woche

- Schritt 2: **Umsetzung planen**

  Je konkreter die Planung gestaltet wird, desto leichter fällt die Ausführung.

Wann?
....

Wo?
....

....

Wie?
....

▶ Schritt 3: **Unterstützung suchen**

Wer kann mir bei der Umsetzung meines Ziels helfen?
(Sportvereine, Freunde/Bekannte, Gesundheitskurse, ...)

....

Welche Hindernisse können mich von der Umsetzung meines Ziels abhalten?

....

....

....

Welche Strategien kann ich nutzen, um diese Hindernisse zu überwinden?

....

....

....

▶ Schritt 4: **Erfolgskontrolle**

Auf den folgenden Seiten finden Sie ein **Tagebuch für körperliche Aktivitäten**, um Ihre Erfolge zu beobachten.
Dieses Tagebuch können Sie als Kopiervorlage nutzen.
Eine Druckvorlage für solch ein Tagebuch sowie weitere Informationen und Hilfestellungen finden Sie auch im Downloadbereich des **Onlineportals**

www.herzwegweiser.de
Berlin & Brandenburg

Woche vom .... bis zum ....

	Aktivität	Dauer in Min.
**Montag**		
**Dienstag**		
**Mittwoch**		
**Donnerstag**		
**Freitag**		
**Samstag**		
**Sonntag**		

Woche vom .... bis zum ....

	Aktivität	Dauer in Min.
Montag		
Dienstag		
Mittwoch		
Donnerstag		
Freitag		
Samstag		
Sonntag		

Herausgeber:

Charité – Universitätsmedizin Berlin
Institut für Medizinische Soziologie und Rehabilitationswissenschaft
Abteilung Rehabilitationsforschung

Luisenstraße 13 A
10117 Berlin

Telefon 030 450 517 109 | www.charite.de

CHARITÉ
UNIVERSITÄTSMEDIZIN BERLIN

www.herzwegweiser.de
Berlin & Brandenburg

Mit freundlicher Unterstützung von:

Deutsche Rentenversicherung
Berlin - Brandenburg

Anzeichen für einen Herzinfarkt

Bei einem oder mehreren der folgenden Anzeichen rufen Sie sofort den Notruf 112 !

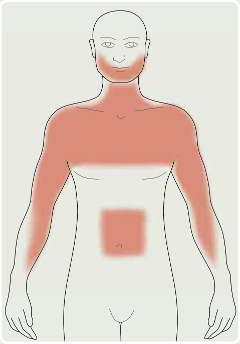

- Starke Schmerzen im Herz- oder Brustbereich, teilweise ausstrahlend in andere Körperregionen wie Arme, Oberbauch, Rücken o. ä.

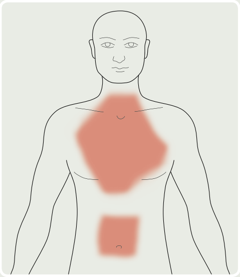

- Engegefühl oder stark brennendes Gefühl im Herz- oder Brustbereich
- Angstgefühl, Angstschweiß, blasse/fahle Haut
- Übelkeit, Erbrechen, Atemnot oder andere unspezifische Beschwerden in zuvor noch nie erlebtem Ausmaß

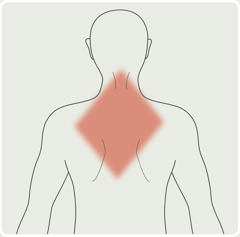

Rettungsdienst: 112
